# The Bioavailability of Glycyrrhizinic Acid Was Enhanced by Probiotic *Lactobacillus rhamnosus* R0011 Supplementation in Liver Fibrosis Rats

**DOI:** 10.3390/nu14245278

**Published:** 2022-12-11

**Authors:** Huifang Li, Jing Wang, Yifan Fu, Ke Zhu, Zhiling Dong, Jinjun Shan, Liuqing Di, Shu Jiang, Tianjie Yuan

**Affiliations:** 1 School of Pharmacy, Nanjing University of Chinese Medicine, Nanjing 210023, China; 2 Jiangsu Key Laboratory of Pediatric Respiratory Disease, Nanjing University of Chinese Medicine, Nanjing 210023, China; 3 Jiangsu Engineering Research Centre for Efficient Delivery System of TCM, School of Pharmacy, Nanjing University of Chinese Medicine, Nanjing 210023, China

**Keywords:** glycyrrhizinic acid, probiotics, oral drugs, bioavailability, gut microbiota

## Abstract

Glycyrrhizinic acid (GL) is clinically applied to treat liver injury, and the bioavailability of orally administered GL is closely related to the gut microbiota. Therefore, the dysbiosis of gut flora in liver injury could significantly influence GL bioavailability. Still, less is known about the impact of probiotic supplementation on the bio-absorption process of oral medication, especially under a pathological state. Herein, probiotic *L. rhamnosus* R0011 (R0011) with a high viability in the harsh gastrointestinal environment was selected, and the effect of R0011 on the GL bioavailability in rats was investigated. Four groups of rats (*n* = 6 per group) were included: the normal group (N group), the normal group supplemented with R0011 (NLGG group), CCl4-induced chronic liver injury model (M group), and the model group supplemented with R0011 (MLGG group). Our results showed that liver injury was successfully induced in the M and MLGG groups via an intraperitoneal injection of 50% (*v*/*v*) CCl4 solution. Healthy rats supplemented with R0011 could increase the bioavailability of GL by 1.4-fold compared with the normal group by plasma pharmacokinetic analysis. Moreover, the GL bioavailability of MLGG group was significantly increased by 4.5-fold compared with the model group. R0011 directly improved gut microbial glucuronidase and downregulated the host intestinal drug transporter gene expression of multidrug resistance protein 2 (MRP2). More critically, R0011 restored the gut microbiota composition and regulated the metabolic function, significantly enhancing the microbial tryptophan metabolic pathway compared with the pathological state, which may indirectly promote the bioavailability of GL. Overall, these data may provide possible strategies by which to address the low bioavailability of traditional medicine through probiotic intervention.

## 1. Introduction

Oral administration is one of the most convenient and safest approaches for drug delivery [1], mainly applied in Chinese herbal medicine utilization [2]. The drug bioabsorption process is traditionally associated with drug physicochemical properties, including drug solubility, polarity, permeability, and stability [3]. Moreover, orally administered drugs are usually absorbed in the gut to enter the circulation system. The gut microbiota, as an “important organ”, is closely correlated with the host metabolism, infection, immune system, brain function, tumors, etc. [4,5]. In addition, the gut microbiota has a chance to come into contact with oral drugs in the gut. Therefore, the gut microbiota acting as a bioreactor may play an important role in the bioabsorption of oral medicines [6].

The gut microbiota can directly transform the parent drugs into bioactive components using intestinal microbial enzymes. Zimmermann has proved that the chemical structures of 271 oral drugs were modified by intestinal microbial enzymes through oxidation, reduction, acetylation, and glucuronidation [7]. Additionally, the gut microbiota can also indirectly regulate drug bioabsorption. Bacterially derived lipopolysaccharide (LPS) can downregulate the drug transporter expression of the sodium-dependent vitamin C transporters SLC23A1 and SLC23A2 and inhibit the intestinal uptake of ascorbic acid [8]. It has been reported that antibiotic-dependent changes in the gut microbiota might lead to significant differences in the transformation, degradation, and bioavailability of orally supplied drugs [9]. Yang et al. (2021) found that antibiotic intervention affected the biotransformation of procyanidin A2 through the gut microbiota, resulting in a 2327.21–6.27-fold decrease in its bioavailability [10]. Hence, modulation of the gut microbiota is a potential strategy for improving oral drug bioavailability. 

Probiotics are live microorganisms that, when administered in adequate amounts, confer a health benefit on human health [11], which has attracted lots of attention. *L. rhamnosus* is one of the most widely used probiotics, with the characteristics of resistance to acid and the ability to adhere to the intestinal layer [12]. Recently, it has been reported that oral supplementation with *L. rhamnosus* (JB-1) can alleviate stress-related disorders [13]. *L. rhamnosus* GG prevented liver injury and fibrosis in mice by suppressing bile acid de novo synthesis and enhancing bile acid extraction [14]. The strain *L. rhamnosus* R0011 was isolated from a dairy culture in 1976, and it has been shown to have a beneficial effect in animal models and human clinical trials [15]. *L. rhamnosus* R0011 can adhere to human epithelial cells [16], helping to maintain the barrier function. A recent study showed that *L. rhamnosus* R0011 can modulate intestinal epithelial cells’ (IECs) response to innate immune stimulants by secreting bioactive molecules using established rodent and human IEC models [17]. However, the direct link between probiotics and the gut microbiota composition and metabolic function has not been well-established. 

Glycyrrhizinic acid (GL) is one of the most widely used Chinese herbal medicines in clinical practice [18]. GL possesses multiple biological activities, including liver protection, anti-inflammation, antioxidation, and antiviral activity [19,20]. According to the latest research, GL showed an efficient anti-coronavirus effect in vitro [21]. Nevertheless, the bioavailability of orally administered GL was extremely low due to the considerable relative molecular weight and polarity. After oral ingestion, GL is first hydrolyzed to glycyrrhetinic acid (GA) by the gut microbiota and then absorbed into the blood to exert a therapeutic effect. Hence, the gut microbiota is directly involved in the pharmacokinetic parameters of orally administered GL [22]. Moreover, the liver injury can induce intestinal dysbiosis [14], and the disorder of gut microbiota composition and metabolic function can significantly influence the bioavailability of GL. However, less is known about the impact of probiotic supplementation on the bioabsorption process of oral drugs, especially under a pathological state. 

Our previous study demonstrated that GL was partially transformed to GA by *L. rhamnosus* R0011 in vitro [23]. However, the specific effect of *L. rhamnosus* R0011 on orally administered GL in vivo has not been explored. In the current study, the human probiotic *L. rhamnosus* R0011 was screened and applied to investigate the positive effect on the pharmacokinetics of orally administered GL, especially in the liver injury status. Furthermore, the underlying mechanism of bioavailability enhancement through supplementation with *L. rhamnosus* R0011 was elucidated via 16S rRNA sequencing and untargeted metabolomics. The deep investigation of the relationship between gut microbes and oral drug bioavailability could contribute to the understanding of inter-individual variation in bioavailability, which may promote individual treatment and better clinical therapeutic efficacy.

## 2. Materials and Methods 

### 2.1. Chemicals and Reagents

GL (≥98% purity) and GA (≥98% purity) were acquired from Yuanye Bio-Technology Co. (Shanghai, China). Acetonitrile and methanol were provided by Merck KGaA (Darmstadt, Germany). Formic acid (FA) was obtained from ACS (Wilmington, DE, USA), and CCl_4_ and olive oil were bought from Macklin Biochemical Technology Co. (Shanghai, China). Trypsin (1:250) and pepsin (1:10,000) were purchased from Solebo Technology Co. (Beijing, China). Alanine aminotransferase (ALT) and aspartate aminotransferase (AST) assay kits were obtained from Jiancheng Bioengineering Institute (Nanjing, China). 

### 2.2. Culture and Screening of Experimental Strains 

Lactobacillus paracasei 2001-12, Lactobacillus salivarius JCM 123, Lactobacillus plantarum NCIMB 8826, Lactobacillus acidophilus JCM 1132, Lactobacillus bulgaricus KLDS 1.0207, and Lactobacillus rhamnosus R0011 were obtained from the China General Microbiological Culture Collection Center (CGMCC) under accession numbers 1.2884, 1.1881, 1.69712, 1.1878, 1.16075, and 1.8882. Lactobacillus was cultured in Man-Rogosa-Sharpe (MRS) broth (Solarbio, Beijing, China) in an anaerobic environment (37 °C) for 24 h, and then the bacteria were collected via centrifugation (8000 rpm for 10 min at 4 °C). The precipitate was washed twice and resuspended in sterile normal saline at a final concentration of 1 × 10^9^ CFU/mL for further use [24].

An total of 100 μL of six lactobacillus liquid cultures was added to 10 mL of simulated gastric juice and simulated intestinal juice. The simulated gastric juice and simulated intestinal juice were prepared according to the methods of the United States Pharmacopoeia. The mixtures of simulated gastric juice and bacterial liquid were incubated at 37 °C for 3 h, and then the viable bacteria were counted on MRS agar. The mixtures of simulated intestinal juice and bacterial liquid were incubated at 37 °C for 4 h, and then the viable bacteria were counted on MRS agar [25].

A dose-dependent (1–20 mg/mL of GL) study was designed to observe the effect of GL on the growth of *L. rhamnosus* R0011. The master liquor of GL was prepared at the concentration of 200 mg/mL and diluted to 0.5, 2.5, 5, 10, 15, and 20 mg/mL using ddH_2_O. After that, the sample was added to 96-well plates, with each well containing 100 μL of diluted bacterial solution (1 × 10^3^ CFU/mL) and 100 μL GL solutions of different concentrations (1–20 mg/mL). For the control group, 100 μL of MRS broth medium and 100 μL diluted GL solutions of different concentrations were added. The bacterial turbidity was measured at OD_600_ after 24 and 48 h of incubation at 37 °C.

### 2.3. Preparation of Animal Models

Male Sprague Dawley (SD) rats (180–220 g) were purchased from Qinglong Mountain experimental animal Co., Ltd. We used flat-bottomed cages with three rats per cage. These rats were fed standard laboratory food and water ad libitum, housed under controlled conditions (light/dark, every 12 h; temperature, 20–22 °C; humidity, 50 ± 10%), and acclimated for one week before the experimental operation. The body weights of rats in different groups were recorded every week. The experiment was approved by the Ethics Committee of Nanjing University of Chinese Medicine (ACU180305) on 12 March 2018. All experimental procedures were strictly performed according to the guidelines of the Experimental Animal Care and Ethics Committee of Nanjing University of Chinese Medicine. 

**Figure 1:**
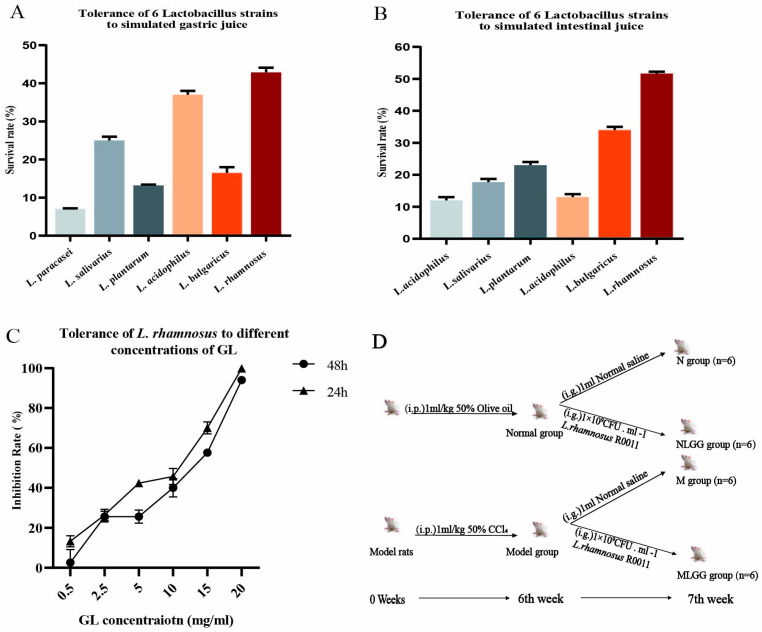
In vitro screening of *Lactobacillus* tolerance in the gastrointestinal environment and animal experimental protocol. (**A**) Tolerance of six *Lactobacillus* species in simulated gastric juice. (**B**) Tolerance of six *Lactobacillus* species in simulated intestinal fluids. (**C**) Tolerance of *L. rhamnosus* R0011 at different concentrations of GL. (**D**) Animal experimental design and protocols for the four groups of rats, the normal group (N group), the normal group supplemented with *L. rhamnosus* R0011 (NLGG group), CCl4 -induced chronic liver injury model (M group), and the model group supplemented with *L. rhamnosus* R0011 (MLGG group).

The rats were randomly divided into four groups (*n* = 6 per group) as follows: the control group (N group), supplementation with *L. rhamnosus* R0011 in the control group (NLGG group), the CCl_4_-induced chronic liver injury model (M group), and supplementation with *L. rhamnosus* R0011 in the model group (MLGG group). The animal experimental protocol is shown in Figure 1D. Chronic liver injury was induced via an intraperitoneal injection of a 50% (*v*/*v*) CCl_4_ solution in olive oil at a dose of 1 mL/kg [26]. In the normal group, CCl_4_ was replaced with olive oil. Three intraperitoneal injections were administered in the first week, followed by twice weekly for six weeks. After the last intraperitoneal injection and a 12 h period of fasting, blood was taken from the orbital vein, and blood samples were centrifuged at 6000 rpm for 10 min to separate the plasma for analysis. The plasma levels of alanine aminotransferase (ALT) and aspartate transaminase (AST) were assessed using assay kits according to the manufacturer’s directions. In the seventh week of the experiment, rats in the N and M groups were gavaged normal saline at a dose of 1 mL, and rats in the other groups were gavaged *L. rhamnosus* R0011 at a dose of 1 mL (1 × 10^9^ CFU/mL) once daily for 1 week. To avoid direct contact with rats’ skin or urine, fresh feces of all rats from the anus were collected into a sterile tube after the last *L. rhamnosus* R0011 and saline treatment [27]. After collection, the feces were quickly stored at −80 °C as early as possible.

### 2.4. Pharmacokinetic Experiments

A Waters HPLC system (Milford, MA, USA) coupled with a Waters Xevo TQD triple quadrupole mass spectrometer (Milford, MA, USA) was used for LC-MS /MS analysis. Chromatographic columns were performed on a ACQUITY UPLC^®^BEH C18, 2.1 × 100 mm, 1.7 µM (Milford, MA, USA), with the column temperature maintained at 40 °C. The mobile phase consisted of (A) 0.4% FA in distilled water and (B) acetonitrile. The following gradient elution scheme was used: 0–1 min, 70–20% A; 1–3 min, 20% A; 3–3.5 min, 20–70% A; 3.5–4.5 min, 70% A. The sample injected into the system for analysis was 10 μL.

After pretreatment with *L. rhamnosus* R0011 or normal saline for 1 week, rats were gavaged a 10 mg/kg dose of GL dissolved in normal saline after fasting with free access to water for 12 h. Blood samples (each 0.5 mL) were collected from the ophthalmic artery plexus of each rat into heparin sodium tubes 0.17, 0.33, 0.5, 0.75, 1, 2, 4, 6, 8, 12, 24, 48, and 72 h after oral administration [26,28]. The plasma was gathered by centrifugation at 6000 rpm for 10 min and stored at −80 °C until further analysis. After blood from the ophthalmic artery plexus was collected, all rats were euthanized with 2% sodium pentobarbital (2 mL/kg, intraperitoneal injection), and then the tissues of the large and small intestine were removed immediately and frozen at −80 °C for qRT-PCR analysis.

Plasma concentration–time profiles were used to evaluate the peak plasma concentration and time of maximum plasma concentration (T_max_) of GL or GA. Other kinetic parameters of the plasma samples were determined using The Drug and Statistics v3.0 (Drug clinical Research Center of Shanghai University of Traditional Chinese Medicine , Shanghai, China )with a non-compartmental statistical model.

### 2.5. Quantitative Real-Time PCR (qRT-PCR)

Total RNA was isolated from intestinal tissues using RNA-easy^TM^ Isolation Reagent and reverse-transcribed into cDNA using the HiScript ^®^ II Q RT SuperMix kit with Gdna Eraser according to the manufacturer’s protocol. Finally, quantitative RT-PCR was executed on a CFX96 real-time PCR detection system (Bio-Rad, Hercules, CA, USA) using the ChamQ SYBR qPCR Master Mix kit. The comparative cycle threshold method quantified the fold-change of the target genes from the internal standard GAPDH. The sequences of the primers are presented in Table 1.

### 2.6. β-Glucuronidase Enzyme Activity Assay in the Feces and Intestinal Tissues

The extraction of crude enzymes from the feces and intestines followed the method of Kim et al. [29]. For the activity assay of fecal and intestinal β-glucuronidase, the reaction mixture (total volume of 0.5 mL), which contained 0.1 mL of 1 mmol/L *p*-nitrophenyl-β-D-glucuronide, 0.3 mL of 0.05 mol/L phosphate buffer (pH 7.2–pH 7.4), and 0.1 mL of the feces and intestinal supernatant, was incubated at 37 °C for 30 min. The reaction was terminated by the addition of 0.5 mL of 10 mmol/L NaOH, and the absorbance was measured at 405 nm (UV–Vis recording spectrophotometer, catalog UV-2401PC; Shimadzu). Enzyme activity was indicated as the amount required to catalyze the formation of 1.0 nmol *p*-nitrophenol/h under standard assay conditions. Specific activity was defined as millimoles per hour per gram of wet feces.

### 2.7. Gut Microbiota Structural Profiling

The E.Z.N.A.^®^ soil DNA Kit (Omega Bio-tek, Norcross, GA, USA) was used to extract total bacterial genomic DNA from fecal samples. The V3 and V4 regions of the bacterial 16S rRNA gene were amplified with primer pairs 338F (5′-ACTCCTACGGGAGGCAGCAG-3′) and 806R (5′-GGACTACHVGGGTWTCTAAT-3′) using an ABI GeneAmp^®^ 9700 PCR thermocycler (ABI, Waltham, CA, USA). According to the standard protocols of Majorbio Bio-Pharm Technology Co., Ltd. (Shanghai, China), purified amplicons from all samples were pooled in equimolar amounts and paired-end-sequenced on an Illumina MiSeq PE300 platform/NovaSeq PE250 platform (Illumina, San Diego, CA, USA). Operational taxonomic units (OTUs) with a 97% similarity cutoff were clustered using UPARSE (version 7.1 http://drive5.com/uparse/, accessed on 8 July 2021) [30], and chimeric sequences were identified and removed. The taxonomy of each OTU representative sequence was analyzed using RDP Classifier (http://rdp.cme.msu.edu/, accessed on 8 July 2021) [31] against the 16S rRNA database (Silva v138), with a confidence threshold of 0.7.

### 2.8. Fecal Metabolic Profiling by UPLC-MS/MS

An total of 50 mg of each fecal sample was pretreated with 400 µL methanol: water (4:1, *v*/*v*) solution. The mixture was processed with a Wonbio-96c high-throughput tissue disruptor (Shanghai Wanbo Biotechnology Co., Ltd., Shanghai, China) at 50 Hz for 6 min, followed by vortexing for 30 s and ultrasound for 30 min at 5 °C. All the samples were placed at −20 °C to precipitate proteins, and then the supernatant was transferred to sample bottles for LC-MS/MS analysis after centrifugation at 13,000× *g* for 15 min at 4 °C.

Chromatographic experiments were carried out on an ACQUITY UPLC BEH C18 column (100 mm × 2.1 mm i.d., 1.7 µm; Waters, Milford, CT, USA), and a UPLC system and a quadrupole time-of-flight mass spectrometer (Triple TOFTM5600+, AB Sciex, Framingham, MA, USA) were used to perform the LC-MS analysis. The column temperature was maintained at 40 °C with a flow rate of 0.4 mL/mins. The mobile phases consisted of 0.1% formic acid in water (solvent A), and 0.1% formic acid in acetonitrile: isopropanol (1:1, *v*/*v*) (solvent B). The gradient of mobile phase B was as follows: 5–20% (0–3 min), 20–95% (3–9 min), 95% (9–13 min), 95–5% (13–13.1 min), and 5% (13.1–16 min). The injection volume was 20 µL.

After UPLC-TOF/MS analyses, the raw data were imported to Progenesis QI 2.3 (Waters, Nonlinear Dynamics, Miford, MA, USA) for peak detection and alignment. Partial least squares discriminant analysis (PLS-DA) was performed in “ropls”. The potential biomarkers were selected using the variable importance in projection (VIP ˃ 1) and statistically significant differences (*p* < 0.05) through a two-tailed *t*-test. Pathway enrichment analysis of the biomarkers was performed on scipy stats (Python package) (https://docs.scipy.org/doc/scipy/, accessed on 8 August 2021) based on the Kyoto Encyclopedia of Genes and Genomes (KEGG) library (https://www.kegg.jp/kegg/pathway.html, accessed on 7 August 2021). The results were analyzed using the Majorbio Cloud Platform (https://www.majorbio.com, accessed on 8 August 2021).

### 2.9. Statistical Analysis

Graphpad prism 9.0 (GraphPad Software, San Diego, CA, USA, https://www.graphpad.com) was used for statistical analyses. All data were analyzed via Student’s *t*-test or ANOVA. The results are expressed as the standard error of the mean (±SEM), and *p* < 0.05 was considered to be statistically significant. Spearman correlation analysis was used in the correlation analysis.

## 3. Results

### 3.1. In Vitro Screening of Lactobacillus Based on Tolerance to the Gastrointestinal Environment

Gastrointestinal tolerance is a vital requirement for practically applying probiotics, and bacterial strains with high survival rates in the harsh gastrointestinal tract environment are essential in order to exhibit beneficial effects [32,33]. In this study, several available *Lactobacillus* probiotics, including *Lactobacillus paracasei* 2001-12, *Lactobacillus salivarius* JCM 1231, *Lactobacillus plantarum* NCIMB 8826, *Lactobacillus acidophilus* JCM 1132, *Lactobacillus bulgaricus* KLDS 1.0207, and *Lactobacillus rhamnosus* R0011, were firstly selected and screened for survival ability in the artificially simulated gastrointestinal environment. As shown in Figure 1A,B, all six *Lactobacilli* showed a certain survival ability. However, *L. rhamnosus* R0011 showed a better performance, with a 42.87% survival rate in the simulated gastric fluid and a 51.67% survival rate in the simulated intestinal fluid. Additionally, GL was reported to have antibacterial bioactivity; therefore, the GL resistance of *L. rhanmosus* R0011 was also investigated. Our results showed that *L. rhanmosus* R0011 possessed a solid tolerance to GL, and the highest concentration of GL reached 20 mg/mL, as shown in Figure 1C. Overall, *L. rhamnosus* R0011 maintained a high viability under the challenging environment, which could guarantee further application.

### 3.2. Effects of L. rhamnosus R0011 on Pharmacokinetics of Glycyrrhizinic Acid (GL)

**Figure 2:**
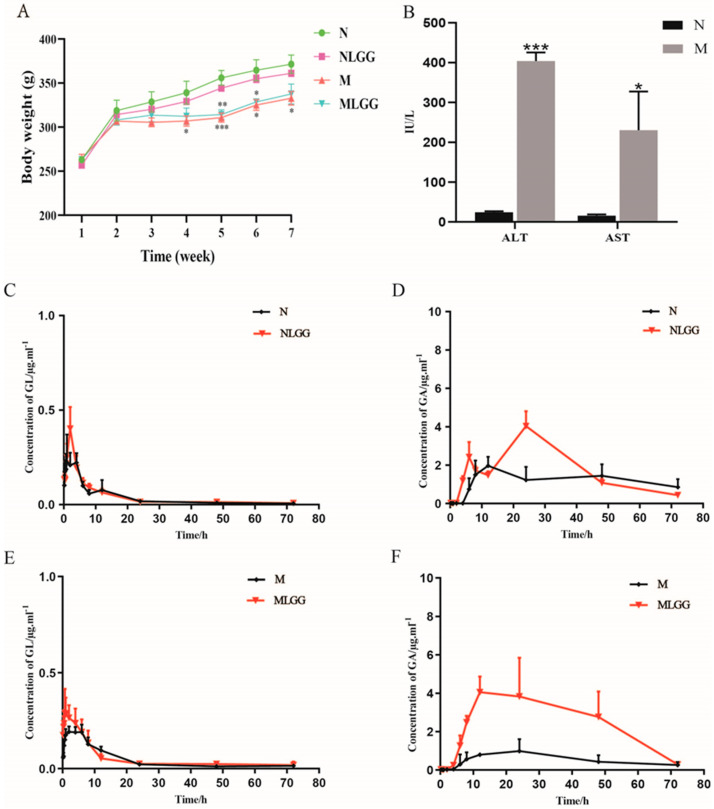
*Lactobacillus rhamnosus* R0011 (*L. rhamnosus* R0011) promoted the bioavailability of glycyrrhizinic acid (GL) under liver fibrosis state. (**A**) The curves of body weight changes of the four group rats. (**B**) ALT and AST in the plasma of rats in the normal and model groups. (**C**) Plasma concentration profiles of GL treated with *L. rhamnosus* R0011 in the healthy group. (**D**) Plasma concentration profiles of glycyrrhetinic acid (GA) treated with *L. rhamnosus* R0011 in the healthy group. (**E**) Plasma concentration profiles of GL treated with *L. rhamnosus* R0011 in the model group. (**F**) Plasma concentration profiles of GA treated with *L. rhamnosus* R0011 in the model group. Data are presented as the mean ± SEM (*n* = 6). The data were analyzed via Student’s *t*-test (* *p* < 0.05; ** *p* < 0.01; *** *p* < 0.001).

To investigate the clinical effect on GL pharmacokinetics, *L. rhamnosus* R0011 was applied under normal and liver injury conditions in Figure 1D. CCl_4_ was used to induce chronic liver fibrosis in rats. Body weight was an essential indicator of rat health status. Figure 2A showed a significant decrease in body weight in M and MLGG groups compared with the normal group since the fifth week. The liver functional biochemical analysis of ALT and AST indicated that liver injury was successfully induced in the CCl_4_ treatment group (Figure 2B). The effect of *L. rhamnosus* R0011 on the pharmacokinetics of orally administered GL was further investigated over a week of intragastric administration. The plasma concentration levels of GL and its metabolite GA were determined in the normal and model groups. Figure 2 and Table 2 summarize the pharmacokinetic parameters of GL and its metabolite GA in the different experimental groups. The AUC value of GL was observed to have no change after *L. rhamnosus* R0011 administration (AUC = 2.98 ± 1.14 µg/mL h) compared with the normal group (AUC = 2.66 ± 0.56 µg/mL h), as shown in Figure 2C. In contrast, the AUC value of GA significantly increased by 1.44 times compared with the normal group after the *L. rhamnosus* R0011 intervention, as shown in Figure 2D.

Furthermore, the addition of *L. rhamnosus* R0011 in the model group was also investigated. A similar tendency was observed in the MLGG group. The AUC value of GL was just slightly improved compared with the model group, as shown in Figure 2E. However, remarkably increased plasma levels of GA (AUC = 181.62 ± 138.60 µg/mL h) were observed after *L. rhamnosus* R0011 supplementation in comparison with the model group (AUC = 39.83 ± 20.49 µg/mL h) (Figure 2F). Our results showed that the accumulative concentration of GA was significantly increased after probiotic intervention, which suggests that *L. rhamnosus* R0011 could dramatically promote the bioavailability of GL, especially under the pathological state.

### 3.3. Effects of L. rhamnosus R0011 on Intestinal Biotransformation of Glycyrrhizinic Acid (GL)

**Figure 3:**
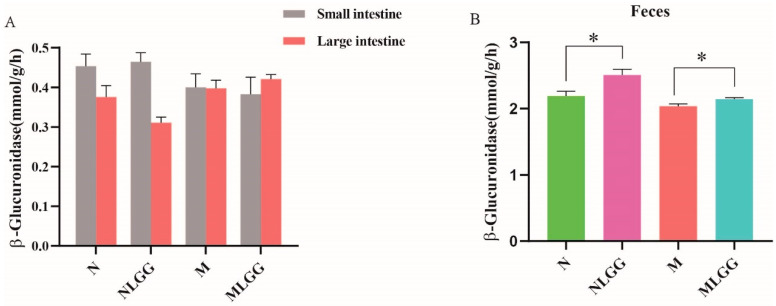
Differences in β-glucuronidase in intestinal tissue extracts and feces in the control group (N group), the control group supplemented with *Lactobacillus rhamnosus* R0011 (*L. rhamnosus* R0011) (NLGG group), the CCl_4_-induced chronic liver injury model (M group), and the model group supplemented with *L. rhamnosus* R0011 (MLGG group). (**A**) β-Glucuronidase in large-intestinal and small-intestinal tissue extracts. (**B**) β-Glucuronidase in feces. The results of the data statistics are expressed as the mean ± SEM (*n* = 6). One-way ANOVA was used for the analysis (* *p* < 0.05).

As glucuronidase is capable of converting GL to GA through the deglycosylation process [34], we examined glucuronidase activity in intestinal tissues as well as in feces. Figure 3A shows that host intestinal metabolic enzyme activities, whether from the tissue extracts of the small intestine or large intestine, were not significantly changed after *L. rhamnosus* R0011 administration. In contrast, the fecal glucuronidase activities were elevated considerably after *L. rhamnosus* R0011 treatment both in the normal and model groups (Figure 3B). These results indicate that supplementation with *L. rhamnosus* R0011 may directly participate in GL biotransformation, which promotes the bioavailability of GL. Treatment with *L. rhamnosus* R0011 could improve the microbial enzyme activities rather than modulating the host intestinal enzyme activities.

### 3.4. Effect of L. rhamnosus R0011 on the Gene Expression of Intestinal Drug Transporters

**Figure 4:**
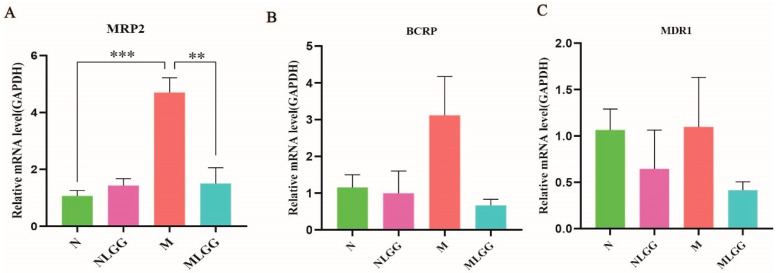
Differences in the relative gene expression of transporter genes in the small intestine in the control group (N group), the control group supplemented with *Lactobacillus rhamnosus* R0011 (*L. rhamnosus* R0011) (NLGG group), the liver cirrhosis group (M group), and the liver cirrhosis group supplemented with *L. rhamnosus* R0011 (MLGG group). (**A**) Multidrug resistance protein 2 (MRP2). (**B**) Breast cancer resistance protein (BCRP). (**C**) Multidrug resistance gene 1 (MDR1). Data are presented as the mean ± SEM (*n* = 6). The data were analyzed via one-way ANOVA (** *p* < 0.01; *** *p* < 0.001).

Intestinal membrane transporters play critical roles in the oral bioavailability of numerous drugs. Therefore, the small-intestinal mRNA levels of drug export pumps, including MDR1, MRP2, and BCRP, related to GA export were investigated after the probiotic intervention. Our results showed that the relative gene expression level of MRP2 was prominently elevated in the model group compared with the normal group, and the trend was reversed after supplementation with *L. rhamnosus* R0011 (Figure 4A). Additionally, *L. rhamnosus* R0011 slightly reduced the relative gene expression of transporters BCRP and MDR1 (Figure 4B,C). These findings show that the downregulation of small-intestinal exporter gene expression by *L. rhamnosus* R0011 treatment might directly contribute to the absorption of GL and GA in vivo.

### 3.5. Structural Modulation of Gut Microbiota by L. rhamnosus R0011 Intervention

**Figure 5:**
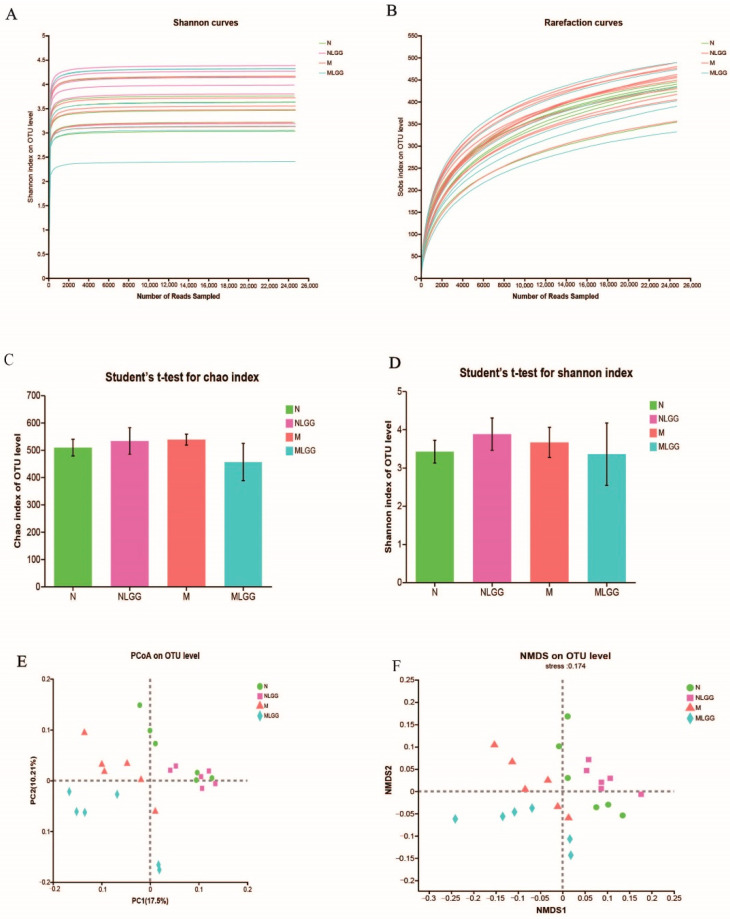
The taxonomic gut microbiota of four groups (control group (N group), control group supplemented with *Lactobacillus rhamnosus* R0011 (*L. rhamnosus* R0011) (NLGG group), liver cirrhosis group (M group), liver cirrhosis group supplemented with *L. rhamnosus* R0011 (MLGG group)). (**A**) Shannon–Wiener curves of samples. (**B**) Rarefaction curves in different groups. (**C**) Chao1 index. (**D**) Shannon index. (**E**) PCoA analysis. (**F**) NMDS analysis (*n* = 6).

Orally administered probiotics could also change the gut microbiota composition. To obtain further insights into the impact of *L. rhamnosus* R0011 on the structure of the gut microbiota, 16S rRNA sequencing of the fecal samples was performed. A total of 1,005,069 high-quality sequences were obtained from 24 samples, with an average of 41,878 reads per sample. According to the tendency of individual rarefaction curves and Shannon–Wiener curves (Figure 5A,B), sequencing data were reasonable and could reflect the microbial information in all samples. The α diversity, as represented by the Chao1 and Shannon indexes, is usually used for evaluating the community richness and diversity. In this study, the Chao index (Figure 5C) and the Shannon index (Figure 5D) showed no significant difference between any two groups. The NLGG group just slightly increased the gut microbiota richness compared with the N group. Principal coordinate analysis (PCoA) and nonmetric multidimensional scaling (NMDS) analysis were performed to evaluate the β diversity among the four groups. As shown in Figure 5E,F, the OTU-based PCoA and NMDS showed differences between microbial communities of the four groups. Nevertheless, most symbols representing the N and NLGG groups were located close to each other. The structural shift among the four groups indicated that *L. rhamnosus* R0011 could be more inclined to change the gut microbiota composition of the M group.

**Figure 6:**
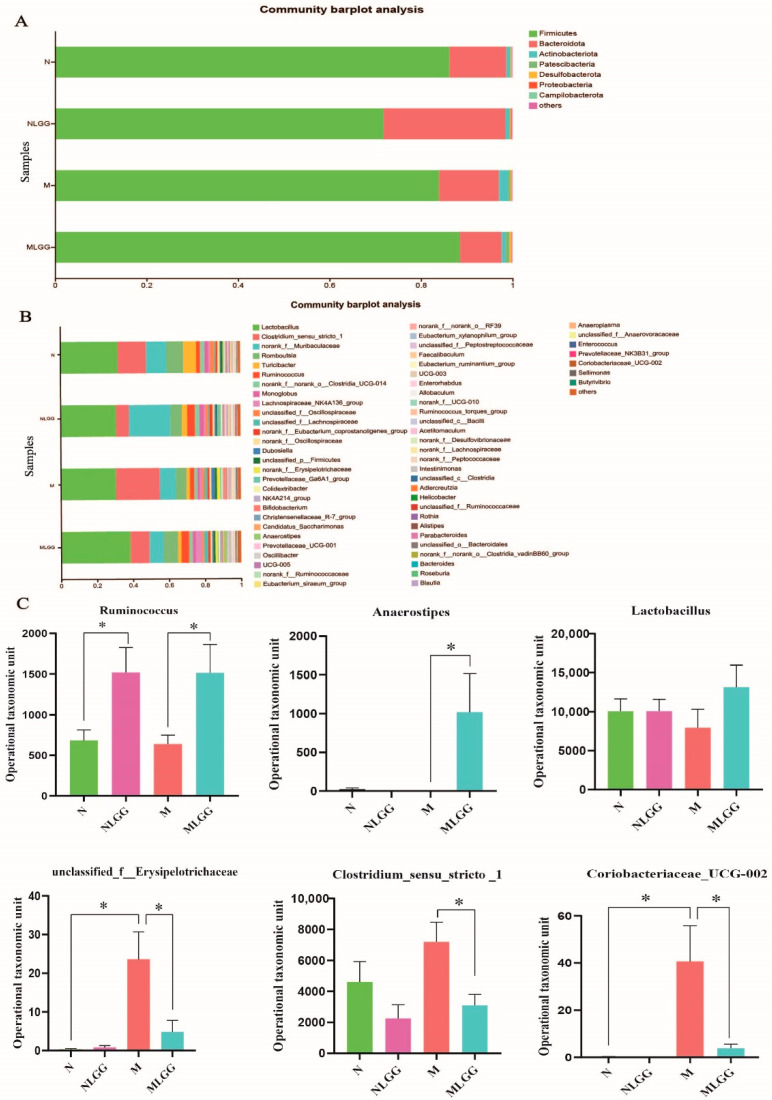
Structural modulation of gut microbiota in the control group (N group), the control group supplemented with *Lactobacillus rhamnosus* R0011 (*L. rhamnosus* R0011) (NLGG group), the CCl_4_-induced chronic liver injury model (M group), and the model group supplemented with *L. rhamnosus* R0011 (MLGG group). (**A**) Phylum-level gut microbiota profile. (**B**) Genus-level gut microbiota profile. (**C**) Alterations in the relative abundance of bacterial taxa. Data are presented as the mean ± SEM (*n* = 6). One-way ANOVA was used to evaluate differences between groups (* *p* < 0.05).

Additionally, we profiled the composition of the gut microbiota at the phylum level in each group through 16S rRNA sequencing analysis. As depicted in Figure 6A, the relative abundance of *Bacteroidetes* and *Actinobacteria* at the phylum level was elevated in the model group compared with the normal group, while *Firmicutes* was reduced to some extent. The disorder of the gut microbiota composition was repristinated after the oral administration of *L. rhamnosus* R0011 under the pathological state. The genus-level distribution of the four groups is illustrated in Figure 6B, showing that the relative abundance of *Clostridium* in the NLGG and MLGG groups decreased after oral administration of *L. rhamnosus* R0011. As shown in Figure 6C, the relative abundance of *Ruminococcus* and *Anaerostipes* markedly increased in the MLGG group compared with the M group. The abundance of *Lactobacillus* increased slightly after the gavage of *L. rhamnosus* R0011 under pathological conditions. In addition, gut microbiota such as *unclassified_f__Erysipelotrichaceae, Clostridium_sensu_stricto _1,* and *Coriobacteriaceae_UCG-002* were massively enriched in the model group and decreased after *L. rhamnosus* R0011 treatment. On the other hand, supplementation with *L. rhamnosus* R0011 will not hugely disturb the richness and composition of a healthy gut microbiota, which could maintain the stability of the gut microbiota in a healthy state.

### 3.6. Effect of L. rhamnosus R0011 on the Modification of Microbial Metabolite Pathways

**Figure 7:**
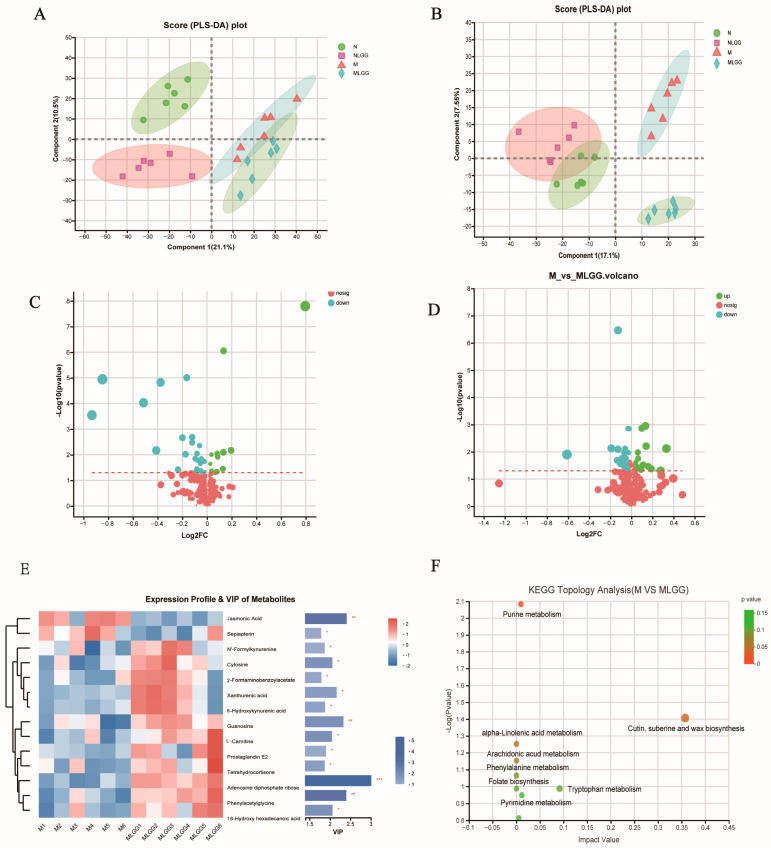
Feces nontarget metabolome analysis in the control group (N group), control group supplemented with *Lactobacillus rhamnosus* R0011 (*L. rhamnosus* R0011) (NLGG group), liver cirrhosis group (M group), and liver cirrhosis group supplemented with *L. rhamnosus* R0011 (MLGG group). (**A**,**B**) PLS-DA score plots under the negative and positive ion modes. (**C**,**D**) Volcano plots of the M and MLGG groups under the negative and positive ion modes. (**E**) Heatmap showing a significant difference in metabolites between M and MLGG (VIP > 1.0, and *p* < 0.05). (**F**) Metabolic pathways between the M and MLGG groups in the KEGG online database (*n* = 6) (* *p* < 0.05, ** *p* < 0.01, *** *p* < 0.001).

The liquid chromatography-mass spectrometry (LC-MS) analytical method was applied to study the fecal metabolome, which partially reflects the functional features of the gut microbiota under probiotic treatment. We built partial least squares discriminant analysis (PLS-DA) models to evaluate the cluster tendencies among the four groups. As shown in Figure 7A,B, distinct clustering of the N, NLGG, M, and MLGG groups was observed in both positive and negative ion modes. In the negative ion mode, the values of R^2^Xcum, R^2^Ycum, and Q^2^cum were 0.316, 0.546, and 0.517. At the same time, values of 0.576, 0.988, and 0.821 were detected in the positive ion mode, indicating a good classification and prediction ability, which could subsequently be used for analyzing the differences among the four groups. Volcano plots (Figure 7C,D) were used to screen biomarkers in both positive and negative ion modes. The variables with VIP ˃ 1 and statistically significant differences (*p* < 0.05) were considered as potential biomarkers.

Based on the result, 14 biomarkers were further analyzed among the model group and the *L. rhamnosus* R0011 treatment group, as shown in Figure 7E. It was observed that the relative abundance of 12 compounds (Adenosine diphosphate ribose, Cytosine, N’-Fomylkynurenine, 2-Formaminobenzoylacetate, Xanthurenic acid, 6-Hydroxykynurenic acid, Prostaglandin E2, Tetrahydrocortisone, Phenylacetylglycine, 16-Hydroxy hexadecanoic acid, Guanosine, L-Carnitine) was significantly increased with the *L. rhamnosus* R0011 supplementation. The identified metabolites were annotated to eight related KEGG metabolic pathways, which were displayed in a metabolome map (Figure 7F). After one week of probiotic intervention, the most significant differences were found in tryptophan metabolism. Evidence has shown that tryptophan metabolites profoundly affect the gut microbial composition, microbial metabolism, and host immune system–intestinal microbiota interactions [35]. Numerous tryptophan metabolites, such as xanthurenic acid and kynurenic acid, have been reported to be aryl hydrocarbon receptor (AhR) ligands. AhR is abundantly present at mucosal surfaces, which are activated to improve intestinal homeostasis and may indirectly affect the bioabsorption of oral drugs [36].

**Figure 8:**
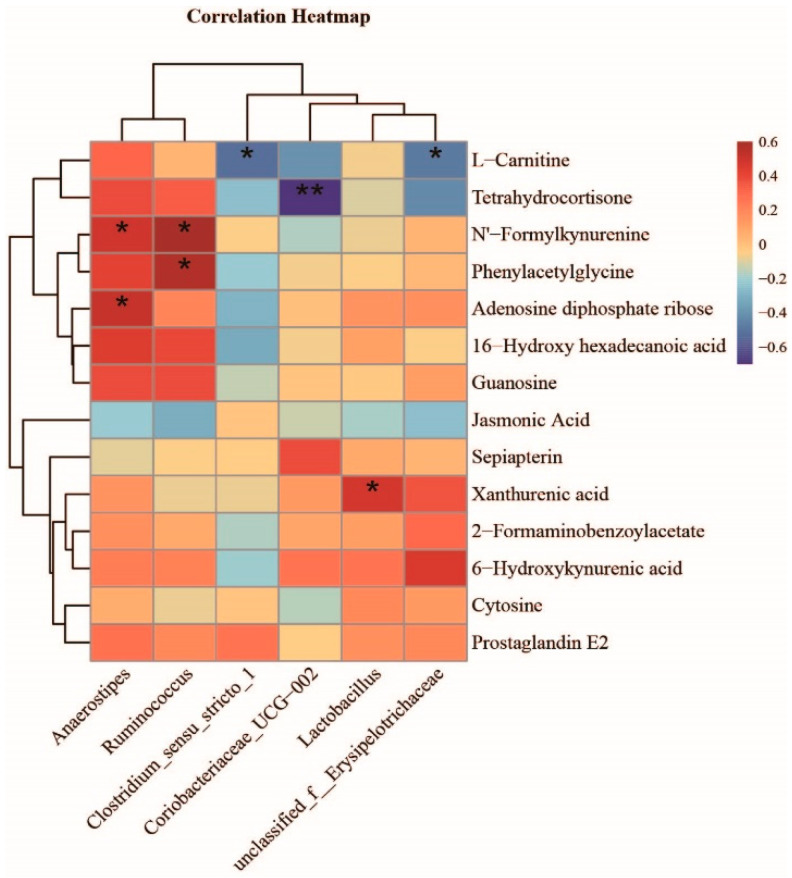
Correlation plot showing the relationship between metabolites from the feces and gut bacteria. Correlation analysis was performed using Spearman’s method (* *p* < 0.05, ** *p* < 0.01).

To determine the functional correlation between the intestinal microbiota and their metabolites, correlation analysis was conducted by calculating Spearman’s correlation coefficient. The resulting metabolic association heatmap (Figure 8) revealed potential correlations between metabolic profiles and the gut microbiome. The correlation analysis showed that *Anaerostipes* had positive associations with Adenosine diphosphate ribose and N’-Formylkynurenine (*p* < 0.05), whereas *Clostridium_sensu_stricto _1* and *unclassified_f__Erysipelotrichaceae* were negatively associated with L-Carnitine (*p* < 0.05). Additionally, *Ruminococcus* was positively correlated with the elevation in N’-Formylkynurenine and Phenylacetylglycine (*p* < 0.05). Of particular note is that *Lactobacillus,* which increased in the MLGG group, was positively correlated with Xanthurenic acid (*p* < 0.05).

## 4. Discussion

Growing evidence has indicated that the gut microbiota plays a crucial role in the pharmacokinetics of orally administered drugs [37]. Recent research has shown that the gut microbiome contributed more than 50% of the metabolism of oral medicines [38], and over 60% of the drug response is closely related to the individual gut microbiota [39]. The concept of pharmacomicrobiomics has attracted considerable attention [40]. Gut microbes can produce various enzymes directly involved in the biotransformation of oral drugs and thereby influence the bioavailability of these drugs. Moreover, the gut microbiota can regulate the host gene expression and modulate the host metabolic function, affecting the drug transport and bioabsorption process. Therefore, the gut microbiota has become a promising target for improving the bioavailability and efficiency of oral drugs.

Most traditional Chinese herbal medicines are clinically applied via oral administration [2,41], and their absorption is inevitably affected by the intestinal microbiota. GL is a well-known pentacyclic triterpenoid glycoside obtained from the traditional Chinese medicine licorice, which has been used as a hepatoprotective agent for chronic hepatitis [42]. GL is poorly absorbed after oral administration due to its polar characteristics, which leads to a low bioavailability and limits its clinical efficacy. In our previous study, *L. murine* was isolated from healthy rats. Supplementation with *L. murine* significantly improved the bioavailability of GL [26]. However, the specific effect of *L. murine* on orally administered GL in vivo has not been explored. Moreover, due to the potential pathogenicity, *L. murine* could not be further used in practical applications. Probiotics have been wildly accepted in our daily life and medical use due to their beneficial effect [43]. However, the effect of probiotics on GL bioavailability has not been investigated. *L. rhamnosus* is one of the most widely studied probiotics, and it has antiallergy [44], antioxidative [45], and gut microflora balancing effects [46]. *L. rhamnosus* R0011 is a food-grade probiotic with a clear genetic background, which has been proven to positively affect human clinical use. It has been suggested that *L. rhamnosus* can produce glycosidases and thus hydrolyze glycosidic compounds [47]. Moreover, our results showed that *L. rhamnosus* R0011 could tolerate the harsh intestinal environment and resist the antibacterial activity of GL, which meets the essential requirement for in vivo research.

In the intestine, GL is deglycosylated into glycyrrhetinic acid (GA) by microbial β-glucuronidase. In the current study, fecal glucuronidase activity was significantly increased in the *L. rhamnosus* R0011-treated group. Recently, *Lactobacillus* has been proven to play an important role in the bioconversion of glycoconjugated phytochemicals by phosphotransferase systems (PTS) in the uptake of carbohydrates and phosphor-glucosidases for the deglycosylation process [48]. Rossi et al. reported that probiotic *Lactobacillus* can produce several glycosyl-hydrolases, and aglycone release can be accelerated by glucosidase [49]. The experimental results support the possibility that *L. rhamnosus* R0011 directly participates in the GL biotransformation process. Accumulated evidence has suggested that drug export pumps, including MDR1, MRP2, and BCRP in the small intestine, play a pivotal role in GL bioavailability. Furthermore, the intestinal drug transporter gene expression of MPR1 and MRP2 was found to be upregulated under inflammatory conditions [50]. Our results showed that supplementary *L. rhamnosus* R0011 could directly downregulate the relative gene expression of MRP2, which may reduce the export of GA that contributes to the improvement of the GA concentration in the blood. Strong evidence exists showing that *L. rhamnosus* reveals pili containing a human mucus-binding protein that promotes strong adhesion interactions with host intestinal epithelial cells [51,52]. Specifically, *L. rhamnosus* R0011 can effectively adhere to intestinal epithelial cells and block the adhesion of various pathogens [53,54]. In this context, we propose that orally administered *L. rhamnosus* R0011 could attach to the GI tract and regulate the host intestinal transporter gene expression.

Intervention with probiotics could further modulate the gut microbiota composition and regulate the metabolic function of the gut microbiota. Our results showed that the relative abundances of *Ruminococcus* and *Anaerostipes* belonging to *Lachnospiraceae* were significantly increased compared with the model group after the *L. rhamnosus* R0011 intervention. T. Akno reported that the gut microbiota *Ruminococcus* sp. PO1-3 can directly metabolize GL to GA by producing glucuronidase [55]. A functional metagenomic study also revealed that *Lachnospiraceae* contains genes encoding glycosidases [28,56,57]. Meanwhile, oral administration of *L. rhamnosus* R0011 can also recover the reduction in *Lactobacillus* caused by liver injury, which may also benefit GL metabolism. In addition, our results showed that the densities of *Coriobacteriaceae*, *Clostridium_sensu_stricto-1*, and *unclassified_f__Erysipelotrichaceae*, which are closely related to liver fibrosis and epithelial inflammation, were remarkably decreased after *L. rhamnosus R0011* administration [58,59]. In short, we hypothesized that oral administration of probiotic *L. rhamnosus* R0011 could recruit more gut microbiota to participate in the GL biotransformation. On the other hand, *L. rhamnosus* R0011 can regulate the gut microbiota dysbiosis and restore the intestinal balance.

According to the analysis of fecal metabolites, it was discovered that the metabolite profile in the pathological state was remarkably changed after supplementation with *L. rhamnosus* R0011. The different metabolites were mainly involved in lipid metabolism (cutin suberine and wax biosynthesis, alpha-Linolenic acid metabolism, or arachidonic acid metabolism) and nucleotide metabolism (purine metabolism or pyrimidine metabolism). In particular, tryptophan metabolism was significantly increased after the probiotic intervention. Tryptophan metabolites such as kynurenine and xanthurenic acid have been reported to bind and activate the aryl hydrocarbon receptor (AhR), which is closely associated with intestinal barrier integrity and mucosal immune homeostasis [36]. It was reported that tryptophan metabolism was impaired under chronic inflammatory diseases [60]. Supplementation with *Lactobacillus reuteri*, with a high AhR ligand-production capacity, could improve gut metabolic homeostasis [61]. Additionally, a state of intact intestinal architecture supports drug absorption [62]. Based on our results, we suggest that supplementation with *L. rhamnosus* R0011 may repair intestinal homeostasis by modulating tryptophan metabolism, which could indirectly improve the bioavailability of GL.

As we know, GL is mainly used in chronic liver injury patients, so *L. rhamnosus* R0011 was applied in normal and liver injury rats to explore the effect of probiotics on GL bioavailability. Supplementation with *L. rhamnosus* R1100 increased the AUC value of GA by 1.4-fold compared with the normal group. Moreover, the AUC value of GA was significantly increased by 4.5-fold compared with the model group by adding *L. rhamnosus R0011*. The different effects of *L. rhamnosus R0011* on the healthy and pathological states also attracted our attention.

Based on our experimental data, the drug transporter gene expression of MRP2 was significantly upregulated under liver injury conditions compared with the normal group. Supplementation with *L. rhamnosus* R0011 significantly downregulated the relative gene expression of MRP2 compared with the M group. However, *L. rhamnosus R0011* did not reduce the relative gene expression of MRP2 compared with the N group, as shown in Figure 4A, mainly due to the expression of MPR2 being kept at a low level under the healthy state. We propose that the enhancement of GL bioavailability in the NLGG group was mainly attributed to the elevation in microbial glucuronidase, which was related to the supplementation with *Lactobacillus* and the increase in *Ruminocoucccus*, as shown in Figure 6C.

It has been reported that the gut microbiota structure of liver injury models was disordered, and the dynamic balance between different microbes was disrupted [14,23]. The gut microbiota disorder in liver injury can significantly influence oral drug absorption. In our study, supplementation with *L. rhamnosus* R1100 in liver injury rats increased microbial glucuronidase and downregulated the gene expression of the drug transporter MRP2. More critically, *L. rhamnosus* R1100 regulated the disorder of the gut microbiota and microbial metabolic function under the liver damage state, which indirectly promoted the bioavailability of GL. Therefore, supplementation with probiotic *L. rhamnosus* R1100 has a more profound effect on the bioabsorption of GL under an irrational state. However, the exact mechanism of the different effects of *L. rhamnosus* R1100 on healthy and pathological conditions still requires further investigation.

## 5. Conclusions

In summary, the probiotic *L. rhamnosus* R0011 tolerated the harsh gut environment and significantly improved the bioavailability of GL in rats, especially under a liver fibrosis state. Our research showed that *L. rhamnosus* R0011 intervention directly improved the microbial glucuronidase activity, as well as downregulating the host drug export gene expression. On the other hand, *L. rhamnosus* R0011 restored the gut microbial composition and repaired intestinal homeostasis, indirectly promoting GL bioabsorption. Overall, these data may provide possible strategies to address the low bioavailability of traditional medicines through probiotic intervention.

## Figures and Tables

**Table 1:** Sequences of primers used for RT-PCR analysis.

Gene	Forward Primer (5′-3′)	Reverse Primer (5′-3′)
MRP2	TTCTGGATCCTCTCGGTCTTATG	ATCTGGAAACCGTAGGAGACGAA
BCRP	CAATGGGATCATGAAACCTG	GAGGCTGATGAATGGAGAA
MDR1	GAGCCCATCCTGTTTGACTG	TGTCTCCCACTCTGGTGTTG

**Table 2:** Plasma pharmacokinetic parameters of glycyrrhizinic acid (GL) and glycyrrhetinic acid (GA) following the oral administration of GL in rats treated with normal saline and *L. rhamnosus* R0011.

Pharmacokinetic Parameters	GL				GA			
	N	NLGG	M	MLGG	N	NLGG	M	MLGG
T max (h)	1.72 ± 1.29	1.53 ± 0.78	4.96 ± 4.14	1.75 ± 1.25	17.33 ± 15.11	16 ± 9.00 *	18.00 ± 6.57	21.33 ± 14.68
Cmax (µg/mL)	0.29 ± 0.11	0.42 ± 0.28	0.24 ± 0.08	0.35 ± 0.07	2.05 ± 0.48	4.22 ± 1.85 *	1.16 ± 0.42	5.93 ± 4.35 ^#^
AUC.(0–t) (µg/mL h)	2.66 ± 0.56	2.98 ± 1.44	3.22 ± 0.86	3.68 ± 0.70	88.57 ± 25.81	127.87 ± 4.56	39.83 ± 20.49	181.62 ± 138.60 ^#^
CLz/F/L· (h kg) ^−1^	17.02 ± 2.72	17.75 ± 7.53	15.64 ± 3.31	11.69 ± 2.43 ^#^	0.35 ± 0.24	0.41 ± 0.16	1.52 ± 1.08	0.17 ± 0.11 ^#^

N, normal group; NLGG, normal group supplemented with *L. rhamnosus* R0011; M, liver cirrhosis group; MLGG, liver cirrhosis group supplemented with *L. rhamnosus* R0011. Data are presented as the mean ± SEM (*n* = 6). Compared with the normal group: * *p* < 0.05, Compared with the model group: ^#^ *p* < 0.05.

## Data Availability

The 16S rRNA sequences have been deposited in the NCBI Sequence Read Archive (SRA) database under BioProject accession no. PRJNA825816.

## References

[1] Han Y., Gao Z., Chen L., Kang L., Huang W., Jin M., Wang Q., Bae Y.H. (2019). Multifunctional oral delivery systems for enhanced bioavailability of therapeutic peptides/proteins. Acta Pharm. Sin. B.

[2] Xu J., Chen H.B., Li S.L. (2017). Understanding the Molecular Mechanisms of the Interplay Between Herbal Medicines and Gut Microbiota. Med. Res. Rev..

[3] Weersma R.K., Zhernakova A., Fu J. (2020). Interaction between drugs and the gut microbiome. Gut.

[4] Dey P. (2019). Gut microbiota in phytopharmacology: A comprehensive overview of concepts, reciprocal interactions, biotransformations and mode of actions. Pharmacol. Res..

[5] Pickard J.M., Zeng M.Y., Caruso R., Núñez G. (2017). Gut microbiota: Role in pathogen colonization, immune responses, and inflammatory disease. Immunol. Rev..

[6] Zhang F., He F., Li L., Guo L., Zhang B., Yu S., Zhao W. (2020). Bioavailability Based on the Gut Microbiota: A New Perspective. Microbiol. Mol. Biol. Rev..

[7] Zimmermann M., Zimmermann-Kogadeeva M., Wegmann R., Goodman A.L. (2019). Mapping human microbiome drug metabolism by gut bacteria and their genes. Nature.

[8] Subramanian V.S., Sabui S., Moradi H., Marchant J.S., Said H.M. (2018). Inhibition of intestinal ascorbic acid uptake by lipopolysaccharide is mediated via transcriptional mechanisms. Biochim. Biophys. Acta Biomembr..

[9] Zhang X., Han Y., Huang W., Jin M., Gao Z. (2021). The influence of the gut microbiota on the bioavailability of oral drugs. Acta Pharm. Sin. B.

[10] Yang S., Zhang Y., Li W., You B., Yu J., Huang X., Yang R. (2021). Gut Microbiota Composition Affects Procyanidin A2-Attenuated Atherosclerosis in ApoE-/-Mice by Modulating the Bioavailability of Its Microbial Metabolites. J. Agric. Food Chem..

[11] Araya M., Morelli L., Reid G., Sanders M.E., Stanton C. (2002). Guidelines for the Evaluation of Probiotics in Food.

[12] Segers M.E., Lebeer S. (2014). Towards a better understanding of Lactobacillus rhamnosus GG-host interactions. Microb. Cell Factories.

[13] Bharwani A., Mian M.F., Surette M.G., Bienenstock J., Forsythe P. (2017). Oral treatment with Lactobacillus rhamnosus attenuates behavioural deficits and immune changes in chronic social stress. BMC Med..

[14] Liu Y., Chen K., Li F., Gu Z., Liu Q., He L., Shao T., Song Q., Zhu F., Zhang L. (2020). Probiotic Lactobacillus rhamnosus GG Prevents Liver Fibrosis Through Inhibiting Hepatic Bile Acid Synthesis and Enhancing Bile Acid Excretion in Mice. Hepatology.

[15] (2011). Beneficial Microbes. Benef. Microbes.

[16] Alemka A., Clyne M., Shanahan F., Tompkins T., Corcionivoschi N., Bourke B. (2010). Probiotic colonization of the adherent mucus layer of HT29MTXE12 cells attenuates Campylobacter jejuni virulence properties. Infect. Immun..

[17] Jeffrey M.P., Strap J.L., Jones T.H., Green-Johnson J.M. (2018). Suppression of Intestinal Epithelial Cell Chemokine Production by Lactobacillus rhamnosus R0011 and Lactobacillus helveticus R0389 Is Mediated by Secreted Bioactive Molecules. Front. Immunol..

[18] Asl M.N., Hosseinzadeh H. (2008). Review of pharmacological effects of Glycyrrhiza sp. and its bioactive compounds. Phytother. Res..

[19] Yu J.Y., Ha J.Y., Kim K.M., Jung Y.S., Jung J.C., Oh S. (2015). Anti-Inflammatory activities of licorice extract and its active compounds, glycyrrhizic acid, liquiritin and liquiritigenin, in BV2 cells and mice liver. Molecules.

[20] Gumpricht E., Dahl R., Devereaux M.W., Sokol R.J. (2005). Licorice compounds glycyrrhizin and 18beta-glycyrrhetinic acid are potent modulators of bile acid-induced cytotoxicity in rat hepatocytes. J. Biol. Chem..

[21] Kuchta K., Cameron S., Lee M., Cai S., Shoyama Y. (2022). Which East Asian herbal medicines can decrease viral infections?. Phytochem. Rev..

[22] Ishida T., Jobu K., Kawada K., Morisawa S., Kawazoe T., Shiraishi H., Fujita H., Nishimura S., Kanno H., Nishiyama M. (2022). Impact of Gut Microbiota on the Pharmacokinetics of Glycyrrhizic Acid in Yokukansan, a Kampo Medicine. Biol. Pharm. Bull..

[23] Yuan T., Wang J., Chen L., Shan J., Di L. (2019). Glycyrrhizic acid improving the liver protective effect by restoring the composition of Lactobacillus. J. Funct. Foods.

[24] Wang Q., Lv L., Jiang H., Wang K., Yan R., Li Y., Ye J., Wu J., Wang Q., Bian X. (2019). Lactobacillus helveticus R0052 alleviates liver injury by modulating gut microbiome and metabolome in D-galactosamine-treated rats. Appl. Microbiol. Biotechnol..

[25] Shi L., Pan R., Lin G., Liang X., Zhao J., Zhang H., Chen W., Wang G. (2021). Lactic acid bacteria alleviate liver damage caused by perfluorooctanoic acid exposure via antioxidant capacity, biosorption capacity and gut microbiota regulation. Ecotoxicol. Environ. Saf..

[26] Yuan T., Wang J., Chen L., Shan J., Di L. (2020). Lactobacillus murinus Improved the Bioavailability of Orally Administered Glycyrrhizic Acid in Rats. Front. Microbiol..

[27] Zhang X., Chen S., Duan F., Liu A., Li S., Zhong W., Sheng W., Chen J., Xu J., Xiao S. (2021). Prebiotics enhance the biotransformation and bioavailability of ginsenosides in rats by modulating gut microbiota. J. Ginseng Res..

[28] Zhou W., Wang H., Zhu X., Shan J., Yin A., Cai B., Di L. (2013). Improvement of intestinal absorption of forsythoside A and chlorogenic acid by different carboxymethyl chitosan and chito-oligosaccharide, application to Flos Lonicerae-Fructus Forsythiae herb couple preparations. PLoS ONE.

[29] Kim J.K., Choi M.S., Jeong J.J., Lim S.M., Kim I.S., Yoo H.H., Kim D.H. (2018). Effect of Probiotics on Pharmacokinetics of Orally Administered Acetaminophen in Mice. Drug Metab. Dispos..

[30] Edgar R.C. (2013). UPARSE: Highly accurate OTU sequences from microbial amplicon reads. Nat. Methods.

[31] Wang Q., Garrity G.M., Tiedje J.M., Cole J.R. (2007). Naive Bayesian classifier for rapid assignment of rRNA sequences into the new bacterial taxonomy. Appl. Environ. Microbiol..

[32] Wu Q., Zhang C., Wa Y., Qu H., Gu R., Chen D., Song Z., Chen X. (2022). Correlation between exopolysaccharide biosynthesis and gastrointestinal tolerance of Lactiplantibacillus plantarum. J. Appl. Microbiol..

[33] Xu H., Wu L., Pan D., Zeng X., Cai Z., Guo Y., Wang W., Wu Z. (2021). Adhesion Characteristics and Dual Transcriptomic and Proteomic Analysis of Lactobacillus reuteri SH23 upon Gastrointestinal Fluid Stress. J. Proteome Res..

[34] Akao T. (2000). Differences in the metabolism of glycyrrhizin, glycyrrhetic acid and glycyrrhetic acid monoglucuronide by human intestinal flora. Biol. Pharm. Bull..

[35] Gao J., Xu K., Liu H., Liu G., Bai M., Peng C., Li T., Yin Y. (2018). Impact of the Gut Microbiota on Intestinal Immunity Mediated by Tryptophan Metabolism. Front. Cell. Infect. Microbiol..

[36] Sun M., Ma N., He T., Johnston L.J., Ma X. (2020). Tryptophan (Trp) modulates gut homeostasis via aryl hydrocarbon receptor (AhR). Crit. Rev. Food Sci. Nutr..

[37] Wilson I.D., Nicholson J.K. (2009). The role of gut microbiota in drug response. Curr. Pharm. Des..

[38] Zimmermann M., Zimmermann-Kogadeeva M., Wegmann R., Goodman A.L. (2019). Separating host and microbiome contributions to drug pharmacokinetics and toxicity. Science.

[39] Haiser H.J., Turnbaugh P.J. (2012). Is it time for a metagenomic basis of therapeutics?. Science.

[40] Saad R., Rizkallah M.R., Aziz R.K. (2012). Gut Pharmacomicrobiomics: The tip of an iceberg of complex interactions between drugs and gut-associated microbes. Gut Pathog..

[41] Liu S.T., Zheng S.W., Hou A.J., Zhang J.X., Wang S., Wang X.J. (2022). A review: The phytochemistry, pharmacology, and pharmacokinetics of Curcumae Longae Rhizoma (Turmeric). World J. Tradit. Chin. Med..

[42] Shen C., Zhu J., Song J., Wang J., Shen B., Yuan H., Li X. (2020). Formulation of pluronic F127/TPGS mixed micelles to improve the oral absorption of glycyrrhizic acid. Drug Dev. Ind. Pharm..

[43] Bron P.A., Kleerebezem M., Brummer R.J., Cani P.D., Mercenier A., MacDonald T.T., Garcia-Ródenas C.L., Wells J.M. (2017). Can probiotics modulate human disease by impacting intestinal barrier function?. Br. J. Nutr..

[44] Basturk A., Isik İ., Atalay A., Yılmaz A. (2020). Investigation of the Efficacy of Lactobacillus rhamnosus GG in Infants With Cow’s Milk Protein Allergy: A Randomised Double-Blind Placebo-Controlled Trial. Probiot. Antimicrob. Proteins.

[45] Li J., Li Q., Gao N., Wang Z., Li F., Li J., Shan A. (2021). Exopolysaccharides produced by Lactobacillus rhamnosus GG alleviate hydrogen peroxide-induced intestinal oxidative damage and apoptosis through the Keap1/Nrf2 and Bax/Bcl-2 pathways in vitro. Food Funct..

[46] Goyal N., Tiwari R.P., Shukla G. (2011). Lactobacillus rhamnosus GG as an Effective Probiotic for Murine Giardiasis. Interdiscip. Perspect. Infect. Dis..

[47] Ku S., You H.J., Park M.S., Ji G.E. (2016). Whole-Cell Biocatalysis for Producing Ginsenoside Rd from Rb1 Using Lactobacillus rhamnosus GG. J. Microbiol. Biotechnol..

[48] Theilmann M.C., Goh Y.J., Nielsen K.F., Klaenhammer T.R., Barrangou R., Abou H.M. (2017). Lactobacillus acidophilus Metabolizes Dietary Plant Glucosides and Externalizes Their Bioactive Phytochemicals. mBio.

[49] Rossi M., Amaretti A., Leonardi A., Raimondi S., Simone M., Quartieri A. (2013). Potential impact of probiotic consumption on the bioactivity of dietary phytochemicals. J. Agric. Food Chem..

[50] Erdmann P., Bruckmueller H., Martin P., Busch D., Haenisch S., Müller J., Wiechowska-Kozlowska A., Partecke L.I., Heidecke C.D., Cascorbi I. (2019). Dysregulation of Mucosal Membrane Transporters and Drug-Metabolizing Enzymes in Ulcerative Colitis. J. Pharm. Sci..

[51] Lebeer S., Claes I., Tytgat H.L., Verhoeven T.L., Marien E., von Ossowski I., Reunanen J., Palva A., Vos W.M., Keersmaecker S.C. (2012). Functional analysis of Lactobacillus rhamnosus GG pili in relation to adhesion and immunomodulatory interactions with intestinal epithelial cells. Appl. Environ. Microbiol..

[52] Kankainen M., Paulin L., Tynkkynen S., von Ossowski I., Reunanen J., Partanen P., Satokari R., Vesterlund S., Hendrickx A.P., Lebeer S. (2009). Comparative genomic analysis of Lactobacillus rhamnosus GG reveals pili containing a human- mucus binding protein. Proc. Natl. Acad. Sci. USA.

[53] Foster L.M., Tompkins T.A., Dahl W.J. (2011). A comprehensive post-market review of studies on a probiotic product containing Lactobacillus helveticus R0052 and Lactobacillus rhamnosus R0011. Benef. Microbes.

[54] Wallace T.D., Bradley S., Buckley N.D., Green-Johnson J.M. (2003). Interactions of lactic acid bacteria with human intestinal epithelial cells: Effects on cytokine production. J. Food Prot..

[55] Akao T. (2000). Competition in the metabolism of glycyrrhizin with glycyrrhetic acid mono-glucuronide by mixed Eubacterium sp. GLH and Ruminococcus sp. PO1-3. Biol. Pharm. Bull..

[56] Gloux K., Berteau O., El Oumami H., Béguet F., Leclerc M., Doré J. (2011). A metagenomic β-glucuronidase uncovers a core adaptive function of the human intestinal microbiome. Proc. Natl. Acad. Sci. USA.

[57] Biernat K.A., Pellock S.J., Bhatt A.P., Bivins M.M., Walton W.G., Tran B.N.T., Wei L., Snider M.C., Cesmat A.P., Tripathy A. (2019). Structure, function, and inhibition of drug reactivating human gut microbial β-glucuronidases. Sci. Rep..

[58] Forbes J.D., Chen C.Y., Knox N.C., Marrie R.A., El-Gabalawy H., de Kievit T., Alfa M., Bernstein C.N., Van Domselaar G. (2018). A comparative study of the gut microbiota in immune-mediated inflammatory diseases-does a common dysbiosis exist?. Microbiome.

[59] Wang J., Ji H., Wang S., Liu H., Zhang W., Zhang D., Wang Y. (2018). Probiotic Lactobacillus plantarum Promotes Intestinal Barrier Function by Strengthening the Epithelium and Modulating Gut Microbiota. Front. Microbiol..

[60] Roager H.M., Licht T.R. (2018). Microbial tryptophan catabolites in health and disease. Nat. Commun..

[61] Natividad J.M., Agus A., Planchais J., Lamas B., Jarry A.C., Martin R., Michel M.L., Chong-Nguyen C., Roussel R., Straube M. (2018). Impaired Aryl Hydrocarbon Receptor Ligand Production by the Gut Microbiota Is a Key Factor in Metabolic Syndrome. Cell Metab..

[62] da Silva Ferreira A.R., Märtson A.G., de Boer A., Wardill H.R., Alffenaar J.W., Harmsen H.J.M., Tissing W.J.E. (2021). Does Chemotherapy-Induced Gastrointestinal Mucositis Affect the Bioavailability and Efficacy of Anti-Infective Drugs?. Biomedicines.

